# Antifibrotic effects of curcumin are associated with overexpression of cathepsins K and L in bleomycin treated mice and human fibroblasts

**DOI:** 10.1186/1465-9921-12-154

**Published:** 2011-11-29

**Authors:** Dongwei Zhang, Chuangfang Huang, Changfu Yang, Renzuo J Liu, Jifeng Wang, Jianzhao Niu, Dieter Brömme

**Affiliations:** 1 Department of Oral and Biological Science, Faculty of Dentistry, University of British Columbia, Vancouver, BC, V6T 1Z3, Canada; 2 Preclinical Medicine School, Beijing University of Chinese Medicine, Beijing, 100029, China

**Keywords:** lung fibrosis, curcumin, cathepsins, collagen, TGF-β1, apoptosis, protease inhibitors.

## Abstract

**Background:**

Lung fibrosis is characterized by fibroblast proliferation and the deposition of collagens. Curcumin, a polyphenol antioxidant from the spice tumeric, has been shown to effectively counteract fibroblast proliferation and reducing inflammation and fibrotic progression in animal models of bleomycin-induced lung injury. However, there is little mechanistic insight in the biological activity of curcumin. Here, we study the effects of curcumin on the expression and activity of cathepsins which have been implicated in the development of fibrotic lung diseases.

**Methods:**

We investigated the effects of curcumin administration to bleomycin stimulated C57BL/6 mice and human fetal lung fibroblasts (HFL-1) on the expression of cathepsins K and L which have been implicated in matrix degradation, TGF-β1 modulation, and apoptosis. Lung tissues were evaluated for their contents of cathepsins K and L, collagen, and TGF-β1. HFL-1 cells were used to investigate the effects of curcumin and cathepsin inhibition on cell proliferation, migration, apoptosis, and the expression of cathepsins K and L and TGF-β1.

**Results:**

Collagen deposition in lungs was decreased by 17-28% after curcumin treatment which was accompanied by increased expression levels of cathepsins L (25%-39%) and K (41%-76%) and a 30% decrease in TGF-β1 expression. Moreover, Tunel staining of lung tissue revealed a 33-41% increase in apoptotic cells after curcumin treatment. These *in vivo *data correlated well with data obtained from the human fibroblast line, HFL-1. Here, cathepsin K and L expression increased 190% and 240%, respectively, in the presence of curcumin and the expression of TGF-β1 decreased by 34%. Furthermore, curcumin significantly decreased cell proliferation and migration and increased the expression of surrogate markers of apoptosis. In contrast, these curcumin effects were partly reversed by a potent cathepsin inhibitor.

**Conclusion:**

This study demonstrates that curcumin increases the expression of cathepsins K and L in lung which an effect on lung fibroblast cell behavior such as proliferation, migration and apoptosis rates and on the expression of TGF-β1 in mouse lung and HFL-1 cells. These results suggest that cathepsin-inducing drugs such as curcumin may be beneficial in the treatment of lung fibrosis.

## Background

Lung fibrosis is accompanied by fibroblast proliferation and excessive extracellular matrix deposition primarily in the form of collagens. This leads to a progressive loss of lung function and ultimately death. Besides anti-inflammatory drugs, there is presently no effective and approved medication available in western countries. However, curcumin, an antioxidant from the spice turmeric, is used as alternative medicine in India and China for various inflammatory conditions and lung related ailments and the NIH is presently funding several clinical studies to evaluate the efficacy of curcumin http://clinicaltrials.gov/ct2/results?term=curcumin. Smith and coworkers, have recently demonstrated that curcumin administration resulted in a significant reduction of lung inflammation and collagen deposition in bleomycin induced lung fibrosis in mice and relate these effects to its anti-proliferative activity on fibroblasts and interference in TGF-β1 mediated signaling pathways [1]. Other laboratories reported similar effects of curcumin in related animal models of lung fibrosis [2-4]. However, the exact mechanism of how curcumin exerts its lung protective activity remains elusive.

Lung fibrosis is also accompanied by abnormal proteolytic activity in lungs. Of particular interest are cysteine cathepsins which are potent collagenases and elastases and which have been implicated in caspase-independent apoptosis pathways [5]. Cathepsins are widely expressed in lung tissue [6,7]. It has been shown that cathepsin K (CatK) deficiency exacerbates lung fibrosis in bleomycin-induced lung injury [8] and that it interferes with normal airway development [9]. On the other hand, increased levels of CatK alleviate excessive extracellular matrix deposition and consequently protect lungs from bleomycin induced lung fibrosis [10]. Furthermore, overexpression of CatK has been detected in human lung fibrosis derived fibroblasts [8,11]. CatK is a very powerful collagenase and elastase [12,13]. We have recently demonstrated that CatK is also involved in the homeostasis of TGF-β1 [9]. On the other hand, TGF-β1 downregulates CatK expression in fibroblasts and favors fibrosis [11]. TGF-β1 also reduces the expression of cathepsin L (CatL) in lung epithelial cell [14]. Accumulating evidence suggests that elevated TGF-β1 levels are a consistent clinical feature of pulmonary fibrosis [15]. Upregulation of TGF-β1 resulted in fibroblast proliferation and differentiation as well as in abnormal collagen deposition [1,16], whereas suppressed activity of TGF-β1 afforded a protection from fibrosis [17,18]. We have previously demonstrated that CatK expression suppresses TGF-β1 expression in mouse lung [9]. To what extent curcumin affects the level of cathepsin expression in lung is unclear. The aim of this study is to investigate the effect of curcumin on cathepsin expression in bleomycin-treated mouse lungs and human lung fibroblasts, and their involvement in fibrosis promoting cell behaviors such as proliferation, migration and apoptosis.

## Materials and methods

### Reagents and Antibodies

Bleomycin was obtained from Haizheng Pharmaceuticals (Zhejiang, China). Curcumin, fetal calf serum (FBS), Dulbecco's Modified Eagle Medium (DMEM), and mouse anti β-actin were purchased from Sigma (Saint Louis, MO, USA), TRIzol Reagent for total RNA isolation was from Invitrogen (Paisley, UK), One Step SYBR^® ^PrimeScript^® ^RT-PCR Kit (DRR066A) for real time RT-PCR from TaKaRa (Dalian, China). TdT-mediated dUTP Nick End-labeling (Tunel) was from Promega (Madison, WI, USA). Antibodies against Bax, Bcl-2 and cleaved caspase-3 were obtained from Cell Signaling Technology (Danvers, MA, USA) and goat polyclonal anti-CatL from Santa Cruz Biotechnology (Santa Cruz, CA, USA), mouse anti TGF-β1 for immunohistochemical staining from sigma, chicken anti TGF-β1 for immunoblot analysis from R&D Systems (Minneapolis, MN, USA), and rabbit anti CatK from ProteinTech Group (Chicago, IL, USA). The peptidyl vinyl sulfone inhibitor LHVS (Mu-Leu-hPh-VS-Ph) has been synthesized as described in [19].

### Bleomycin induced lung fibrosis

9-10 C57BL/6 mice per experimental group, age 8-14 weeks, were used in the fibrosis model. Mice were housed in a barrier facility with specific pathogen-free conditions and all experiments were performed using protocols approved by the Beijing University of Chinese Medicine Animal Care Unit. Briefly, mice were anesthetized and the trachea was minimally exposed via a ventral midline neck incision. Bleomycin sulfate (0.1 ml, 0.025 U/mouse, diluted in sterile saline) or equal volumes of saline as control were slowly instilled intratracheally as previously described [20]. The incision was closed with surgical adhesive, and mice were allowed to recover with free access to food and water. Mice were treated (2^nd ^day after instillation) with curcumin (200 mg/kg) or saline alone daily in 0.5% carboxymethylcellulose by gavage. Mice were euthanized at various time points (at 1, 2, and 4 weeks) after bleomycin instillation. Lung tissues were either flash frozen and then stored in liquid nitrogen for further analysis or perfused and fixed in 10% formalin for at least 24 h at room temperature for immunohistochemical analysis.

### Histochemical and immunohistochemical staining

5 μm sections were used for Hematoxylin and Eosin (H&E) staining to evaluate the severity of lung inflammation [21]. Masson Trichrome staining was performed to assess the degree of fibrosis [22]. Microscopic images were acquired with the 20× objective on a Leica microscope (Leica Microsystems Inc., Wetzlar, Germany). Immunohistochemical analysis of the lung tissue samples was performed following standard procedure. Briefly, endogenous peroxidase activity was blocked by immersion of deparaffinized sections in 3% H_2_O_2 _in methanol for 30 min. Antigen retrieval was performed by steaming slides in 0.01 M citrate buffer (pH 6.0) for 30 min. Slides were blocked with 1% bovine serum albumin for 30 min at room temperature and subsequently incubated at 4°C overnight with antibodies against CatK (1:100) and CatL (1:100), TGF-β1 (10 μg/ml). To identify and exclude non-specific immunoglobulin binding in tissue sections, non-antigen derived IgGs of the same species and at the same concentration as the experimental primary antibodies were used as controls. Subsequently, the slides were incubated with biotin conjugated horse anti-mouse IgG (Pierce, Rockford, IL, USA), goat anti-rabbit (Pierce) for 30 min at room temperature. Then immunoreactions were visualized by using avidin biotin conjugated horseradish peroxidase and 3, 3'-diaminiobenzidine (DAB, Vector Labs, Burlingame, CA, USA) as substrate. Two tissue sections of each specimen were assessed at a 40× original magnification using a Leica microscope and evaluated with Openlab software (PerkinElmer, Waltham, MA, USA).

### Tunel assay

End-labeling of exposed 3'-OH ends of DNA fragments was undertaken with the DeadEnd™ Fluorometric Tunel System as described by the manufacturer. Briefly, the tissue samples on slides were permeabilized with proteinase K, then incubated with fluorescein 12-dUTP and finally stained with propidium iodide. After staining, whole lung was imaged using 5× magnification for examination. Images were quantified using Openlab software (PerkinElmer).

### Real-time RT-PCR

mRNA levels were evaluated by using the TaKaRa One Step real-time RT-PCR assay according to the manufacturer's protocol. Total cellular RNA was extracted from lungs using TRIzol reagent (Invitrogen) according to the manufacturer's instructions. The primer sequences were: β-actin (forward, 5'-AGA GGG AAA TCG TGC GTG AC-3'; reverse, 5'-CAA TAG TGA TGA CCT GGC CGT-3') and CatK (forward, 5'-ACT TGG GAG ACA TGA CCA GTG A -3'; reverse, 5'-TCT TGA CTG GAG TAA CGT ATC CTT TC-3'). To normalize the amount of total RNA present in each reaction, β-actin cDNA was employed as an internal control.

### Human lung fibroblast (HFL-1) culture

HFL-1 cells were a gift from Dr. Clive Roberts, University of British Columbia (ATCC, CCL153). The cells were cultured in DMEM containing 10% FBS, penicillin/kanamycin. Cultures were maintained at 37°C in a humidified atmosphere of 5% CO_2_. The medium was changed every other day. Cells were treated with bleomycin (0.1 mU/ml; dissolved in Phosphate buffered saline, PBS) and/or various concentrations of curcumin. Drug treatments of cells and their controls were performed in DMEM without FBS.

### MTT assay

MTT [thiazolyl blue, 3-(4, 5-dimethylthiazol-2-yl)-2, 5-diphenyltertrazolium bromide, Sigma] was used to determine cell viability and proliferation. At specified time points, the culture medium was removed, and fibroblasts were incubated with 5 mg/ml MTT solution in an incubator at 37°C for 3 h. After removing the medium, 200 μl of DMSO was added to each well of a 96-well plate to solubilize the blue-colored tetrazolium and the plates were then shaken for 5 min. Absorbance at 570 nm was recorded in a Spectramax Plus reader (Molecular Devices, Sunnyvale, CA). Data were expressed in percentage with the untreated samples set to 100%.

### Wound healing assay

HFL-1 cells were evenly seeded into a 6-well plate. Cells were starved for 24 h in serum free DMEM after reaching at least 90% confluence. Three separate parallel scratch wounds were made with a 200 μl pipette tip. After washing twice with PBS, 10% FBS in DMEM medium containing different concentrations of curcumin was added to the cells. Images were taken under phase contrast inverted microscopy at 0 and 24 h. The wound area was measured using Openlab software and represented as percentage of wound closure of the time zero wound area.

### Transmigration assay

HFL-1 cells were cultured in DMEM containing 10% FBS. When cells reached 70% confluence they were starved in DMEM without FBS media for 24 h. Cells were trypsinized and resuspended in serum free media. Then 10^4 ^cells were seeded into the upper level of the insert membrane (Transwell Permeable Supports, Corning, Lowell, MA) with different concentrations of curcumin. The bottom of the insert contained DMEM with 10% FBS. The invasion assay was carried out for 8 h in the tissue culture incubator. After 8 h, inserts were rinsed with PBS and stained with 0.5% crystal violet solution in 25% methanol for 10 min. After washing the inserts by dipping the inserts into distilled water, cells at the top of the inserts were removed with a cotton swab. The membrane was soaked in 1% SDS in PBS overnight. On the second day, 200 μl of buffer were transferred to a 96-well plate and the optical density (OD) was measured at a wavelength of 570 nm in a Spectramax Plus reader. The percentage of migrated cells was calculated based on the ODs of treated and untreated samples.

### Immunoblot assay

Proteins in cell lysates were separated by SDS-PAGE and then transferred to nitrocellulose membranes. Membranes were incubated with appropriate primary antibodies overnight at 4°C. The concentrations of primary antibodies were as follows: Bax (1:10000), BCL-2 (1:2000), cleaved caspase-3 (1:1000), β-actin (1:30000), CatL (1:100), TGF-β1 (1:850), CatK (1:2000). Blots were then washed with 0.05% Tween in PBS and incubated for 1 h at room temperature with horseradish peroxidase-conjugated secondary antibodies. Serial images of the blots were captured and analyzed using the Chemigenius bioimaging system (Syngene, Cambridge, UK). All values were normalized to expression of the corresponding β-actin in the same membrane. Pixel densities were corrected for background staining in the same membrane.

### Statistical analysis

Results are expressed as means ± SE for all mouse related experiments. All other results are displayed as means ± SD. All image analyses were performed by two observers blinded to the group status. The significance of differences of the mean values was calculated using parametric ANOVA. A *p *value of less than 0.05 was considered significant.

## Results

### Expressions of CatK and L in response to curcumin treatment in lungs with bleomycin induced fibrosis and HFL-1 cells

**Figure 1:**
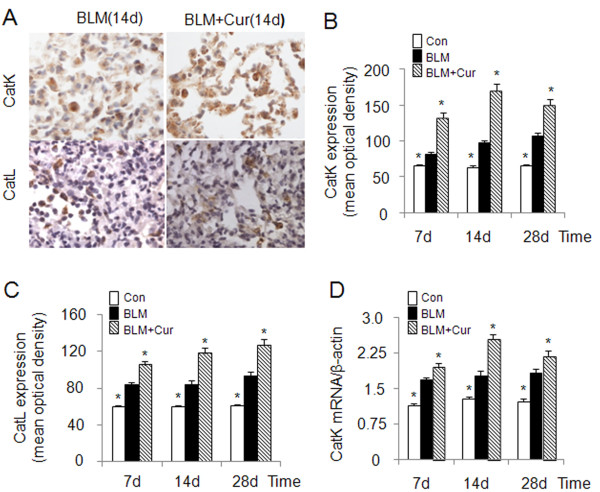
**Effect of curcumin on the expression of cathepsins K and L in mouse lungs of bleomycin induced fibrosis**. Representative images (A) of immunohistochemical staining and quantification (B and C) of the expression of CatK and L in curcumin treated bleomycin induced mice show that expressions of CatK and L were increased at 7d, 14 d and 28 d. Real time PCR results (D) also show RNA levels of CatK were increased after curcumin and bleomycin treatment at the indicated time points. For each time point 9-10 mice were evaluated. All data represent means ± SE (* *p *< 0.05, all compared to bleomycin treated group only; Con: control, BLM: bleomycin, Cur: curcumin).

Cathepsins are involved in human lung development [6] and are transiently upregulated in bleomycin induced lung injury during the regeneration phase [23]. CatK, one of most efficient collagenases [13], has been shown to reduce collagen deposition in lungs of mice with bleomycin induced lung fibrosis when overexpressed [10]. Considering the known anti-fibrotic activity of curcumin in lung [24], we evaluated the effect of curcumin on the expression of CatK and L in bleomycin-induced lung fibrosis. As shown in Figure 1A-C, CatK protein expression in curcumin treated mice was increased by 63%, 76% and 41% at 7d, 14d and 28d, respectively and that of CatL by 25%, 39% and 38% when compared to bleomycin treated mice. Further, at 7d, 14d and 28d, CatK mRNA levels (Figure 1D) in the curcumin treated group also increased by 17%, 44% and 20%, respectively. It should be noted that bleomycin treatment causes maximal fibrosis already after one week.

**Figure 2:**
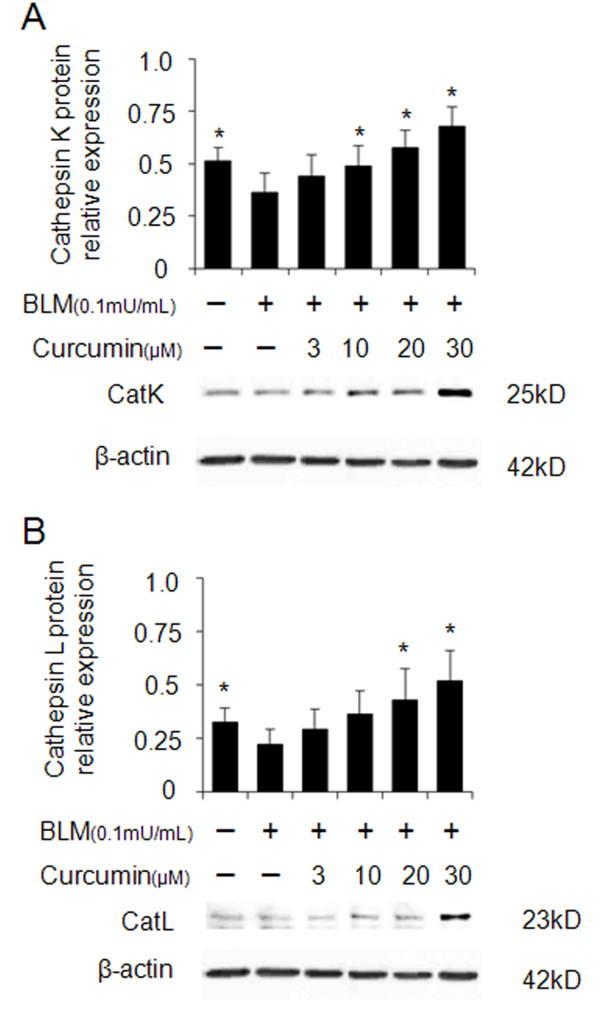
**Overexpression of cathepsins K and L in curcumin treated bleomycin stimulated human lung fibroblasts (HFL-1 cells)**. Representative images (A and B) of immunoblots and measurements show that expressions of CatK and L were dose-dependently increased by curcumin. All data represent means of three independent experiments ± SD and represent percentage cathepsin expression compared to β-actin as control (* *p *< 0.05, all compared to bleomycin treated group only).

Similar to the expression of CatK and L in the lung tissue, the expression of both enzymes was also increased in human fetal lung fibroblast (HFL-1) cultures after treatment with curcumin. As shown in Figure 2A and 2B, CatK and L expression increased in the presence of curcumin in a dose-dependent manner after treatment of the cells with bleomycin. Treatment with 30 μM of curcumin resulted in an about 100% increase in the expression of CatK and L.

### Effect of curcumin on collagen deposition in the lung and on lung fibroblast proliferation and migration

**Figure 3:**
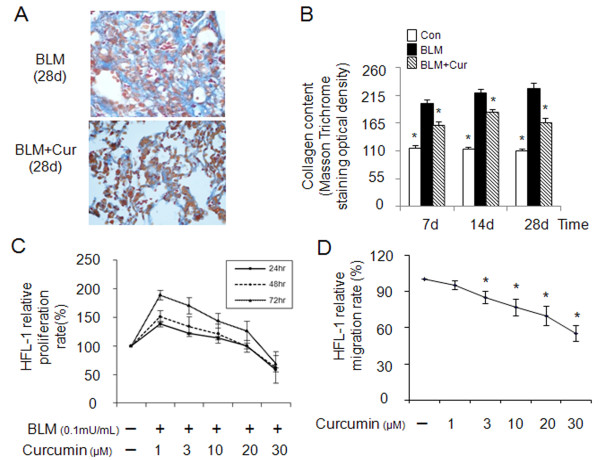
**Effects of curcumin on collagen deposition in fibrotic lungs of mice (bleomycin-induced) and HFL-1 cell proliferation and migration**. Representative images of Masson trichrome staining (A) and quantitative image analysis (B) of collagen content in the lungs of 9-10 mice/group display a reduction of collagen by curcumin. MTT assay (C) shows curcumin dose-dependent decrease of proliferation of HFL-1 cells in the presence of bleomycin (0.1 mU/ml) at 24, 48 and 72 h. Cell migration rates (D) of HFL-1 cells were also decreased by curcumin. All the cell-related data represent means of three independent experiments ± SD. The Masson trichrome staining evaluation is based on the image analysis of two tissue section per mouse and data represent means ± SE (* *p *< 0.05, all compared to bleomycin treated group only).

Bleomycin-induced lung fibrosis is strongly associated with extracellular matrix production and deposition. When compared to untreated control mice, bleomycin-treated animals showed an almost 100% increase in lung collagen deposition 7 days after the initial bleomycin instillation (Figure 3A and 3B). The collagen content further increased to 113% at 28d. Oral application of curcumin (200 mg/kg/d), beginning two days after the bleomycin treatment, reduced the deposition of collagen by 21% after 7d, 17% after 14d and 29% after 28d.

As fibroblasts represent the major cell type in lung fibrotic tissue, we determined the effect of curcumin on the proliferation and migration rates of HFL-1 cells. HFL-1 cells were exposed to bleomycin (0.1 mU/ml) and stimulated for 24, 48 and 72 h. Curcumin revealed a dose-response related inhibition of cell proliferation (Figure 3C) whereas bleomycin increased the proliferation rate by almost 100%. The anti-proliferative effect became significant at curcumin concentrations equal or higher than 10 μM. At 30 μM, the proliferation rates were suppressed below the rate observed in the absence of bleomycin.

Fibroblasts migration may accelerate fibroblast foci formation and contribute to the development of lung fibrosis. Thus, we determined the effect of curcumin on transmigration rates of HFL-1 cells. As shown in Figure 3D, curcumin was effective in attenuating dose-dependently fibroblast transmigration. Migration rates were reduced up to 45% at 30 μM of curcumin.

### Effect of curcumin on wound healing and its dependency on cathepsin activities

**Figure 4:**
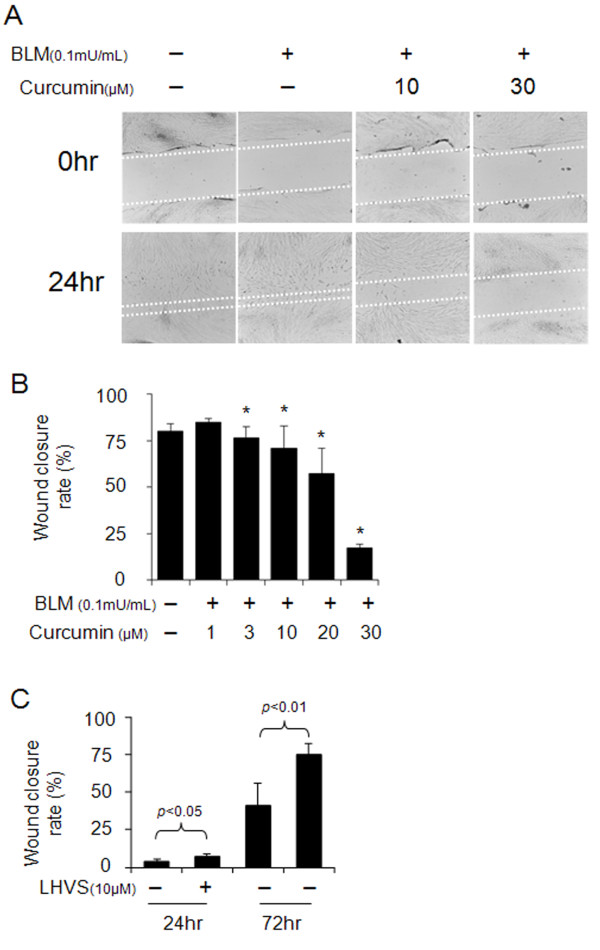
**Effects of curcumin and a cathepsin inhibitor on wound healing in HFL-1 cell scratch model**. Representative images of wound healing assay (A) and its quantification (B) demonstrate the dose-dependent reduction of wound closure rates by curcumin. In contrast, LHVS, a pan-cathepsin inhibitor significantly increased wound closure rates (C). All data represent means of three independent experiments ± SD (* *p *< 0.05, all compared to bleomycin treated group only).

Abnormal wound healing is a main contributor to lung fibrosis. As shown in Figure 4A and 4B, curcumin delayed wound healing in a dose-dependent manner. Wound closure rates were reduced from 75% at 3 μM curcumin to 17% at 30 μM of curcumin concentration. Further, HFL-1 cells were incubated in the presence or absence of 10 μM LHVS, a pan-cathepsin inhibitor, prior to the wound making. After wound making, cells were incubated with curcumin (30 μM) and bleomycin (0.1 mU/ml) with or without LHVS for 24 h and subsequently allowed to grow in drug-free media (10% FBS in DMEM) for another 48 h. As shown in Figure 4C, we found that LHVS can significantly increase the rate of wound closure (p < 0.05 or p < 0.01). These results indicate that curcumin can inhibit proliferation and migration in this assay and that this partly depends on cathepsin activity.

### Effect of curcumin on TGF-β1 expression and its dependency on cathepsin activities

**Figure 5:**
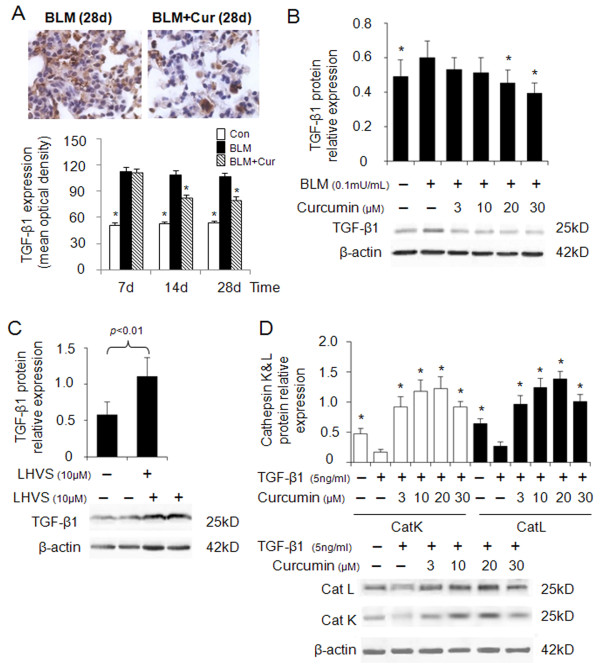
**Effects of curcumin on expressions of TGF-β1, and the effect of TGF-β1 on cathepsins K and L expression**. Representative immunohistochemical images and image analysis (A) of TGF-β1 expression in bleomycin treated mice (9- 10 mice per group). Curcumin significantly decreased TGF-β1 expressions at 14d and 28d. All data besides the TGF-β1 analysis (means ± SE) represent means of three independent experiments ± SD (* *p *< 0.05, all compared to bleomycin treated group only). (B) Representative immunoblot images and their quantitative analysis display the dose-dependent reduction of TGF-β1 protein content by curcumin in bleomycin treated HFL-1 cells. In contrast, LHVS increased TGF-β1 protein content two-fold (C). Panel D shows a 7 and 5-fold increase in the expressions of CatK and L in TGF-β1 treated HFL-1 cells when exposed to curcumin (20 μM) whereas TGF-β1 alone significantly decreased cathepsin expression. All data represent means of three independent experiments ± SD (* *p *< 0.05, all compared to bleomycin or TGF-β1 treated group only).

TGF-β1 plays a key role in the progression of lung fibrosis and has been shown to be upregulated [25]. Inhibition of TGF-β1 attenuates exacerbation of lung fibrosis [26]. First, we determined TGF-β1 expression in bleomycin induced lung fibrosis in the absence or presence of curcumin. As shown in Figure 5A, 28 days after bleomycin administration, the extent of TGF-β1 expression was decreased by 25% in the presence of curcumin when compared to the absence of curcumin at same time point. To reveal the influence of cathepsin activity and their regulation by curcumin on TGF-β1 expression, we treated bleomycin stimulated HFL-1 cells with curcumin. As shown in Figure 5B, TGF-β1 expression was dose-dependently reduced by curcumin. Further, TGF-β1 expression was increased two-fold by the addition of the pan-cathepsin inhibitor, LHVS, in the presence of curcumin (30 μM) and bleomycin (0.1 mU/ml) (Figure 5C). To further elucidate cathepsin expression in response to TGF-β1 stimulation typical in bleomycin-induced lung fibrosis, we treated HFL-1 cells with a constant amount of TGF-β1 (5 ng/ml) and measured the effect of curcumin on CatK and L expression. The expressions of CatK and L were 2.5-fold lower in the TGF-β1 only treated cells than in the control cells (absence of TGF-β1 and curcumin) (Figure 5D). Addition of curcumin (20 μM) increased the expression of CatK and L 7 and 5-fold, respectively.

Taken together, this may indicate that curcumin-induced upregulation of cathepsins reduces the TGF-β1 content in fibroblasts and lung tissue.

### Effect of curcumin on apoptosis and its dependency on cathepsin activities

**Figure 6:**
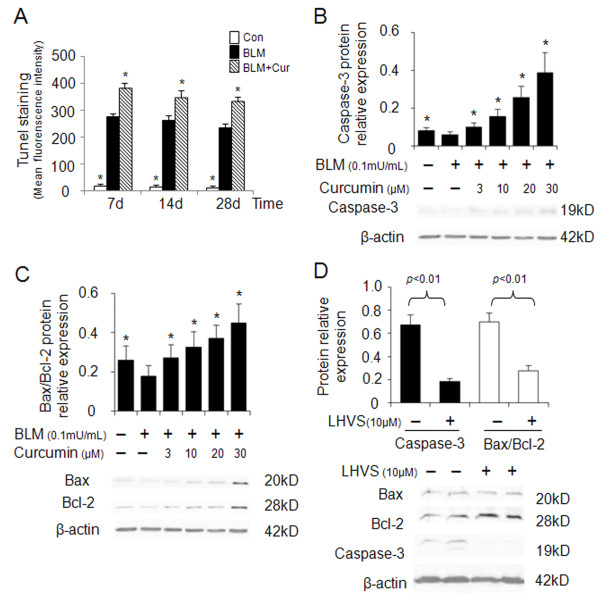
**Effects of curcumin and a cathepsin inhibitor on the expression of apoptosis markers in bleomycin stimulated HFL-1 cells**. Quantitative analysis of Tunel-positive staining in lungs of curcumin treated bleomycin-induced lung injury (A). Representative immunoblot images and their quantitative analysis (B and C) show that curcumin increases caspase-3, Bax/Bcl-2 expression in a dose-dependent manner. LHVS, a pan-cathepsin inhibitor is able to reverse the proapoptotic effect of curcumin as seen by the reduction of caspase-3 expression and the Bax/Bcl-2 ratio in HFL-1 cells (D). All data represent means of three independent experiments ± SD. (* *p *< 0.05, all compared to bleomycin treated group only).

Apoptosis has been associated with both physiological lung homeostasis and pathological lung remodeling [27]. Tissue sections of mouse lungs revealed a 33-41% increase in Tunel positive (apoptotic) cells in curcumin treated mice when compared to control bleomycin-treated littermates only (Figure 6A). This result suggests that apoptosis might contribute to the regeneration of fibrotic lung tissues. In order to investigate the effect of cathepsin expression on promoting fibroblast apoptosis as a response to curcumin administration, we determined the expression of caspase-3 and Bax/Bcl-2 in bleomycin stimulated HFL-1 cells. Figures 6B and 6C show that caspase-3 expression and the ratio of Bax/Bcl-2 in HFL-1 cells were dose-dependently increased by 4.5 and 2.5-fold after 24 h of 30 μM curcumin treatment.

Subsequently, we examined whether the ability of curcumin inducing cell apoptosis was cathepsin dependent, LHVS was utilized to evaluate the alterations of caspase-3, Bax, and Bcl-2 in bleomycin treated HFL-1 cells. HFL-1 cells exposed to bleomycin were treated with 30 μM curcumin in the presence or absence of LHVS. After 24 h, caspase-3 expression was investigated by immunoblotting and immunofluorescence (data not shown). Figure 6D reveals that LHVS significantly inhibited caspase-3 expression (p < 0.05). Accordingly, the ratio of Bax/Bcl-2 was reduced by LHVS as well (p < 0.05). These data indicate that curcumin is proapoptotic and that the curcumin-dependent upregulation of cathepsins is directly contributing to increased apoptosis rates.

## Discussion

Curcumin has been extensively studied as a potential drug for the treatment of lung fibrosis. Most of the recent research indicates that the mechanism of blocking fibrosis by curcumin is related to decreasing collagen accumulation in the lung [1] and to anti-oxidant [2,28-30] and anti-inflammatory activities [24]. *In vitro*, curcumin has the ability of inducing scleroderma fibroblast apoptosis [31], protecting rat lung epithelial cells from quartz particle-mediated cytotoxic and inflammatory effects [30], inhibiting lung fibroblast proliferation [1], blocking the TGF-β1 signaling cascade [32], and upregulating heme oxygenase-1 [33] in renal cells. No data, however, have been reported to demonstrate a curcumin-mediated effect on cathepsin expression in bleomycin induced lung fibrosis as a pathway to control extracellular matrix accumulation, cell proliferation, and apoptosis. Here, we show that curcumin increases CatK and L expression *in vivo *and *in vitro *and that the observed effects on lung fibroblast cell proliferation, migration and apoptosis as well as the expression of TGF-β1 are linked to cysteine cathepsin activities. It should be noted that we also observed an increase in cathepsin B and S expression (albeit to a lesser degree than cathepsins K and L) in lung tissues after curcumin treatment (data not shown).

Bleomycin causes an increased expression of TGF-β1 in activated fibroblasts as shown in Figure 5. Increased TGF-β1 levels promote ECM production and deposition by inducing fibroblast differentiation into myofibroblasts [34,35] and decreasing the expression of ECM degrading proteases such as CatK in these cells [11]. CatK is a highly potent collagenase and elastase and its downregulation exerts a further increase in ECM accumulation in fibrotic lungs as shown in CatK-deficient mice [8]. On the other hand, it has been reported that CatK is overexpressed in human lung fibrosis and silica-induced fibrosis which has been interpreted as a countermeasure to the increased ECM deposition [8,11]. In support of these findings, CatK overexpressing transgenic mice revealed a low degree of fibrosis in response to bleomycin challenge when compared with wild type mice [10]. Here, we show that the antifibrotic activity of curcumin is accompanied by about 2-fold increases in CatK and L expression. An anti-fibrotic effect of cathepsin overexpression can have multiple causes. First, increased tissue levels of proteases such as CatK will increase ECM degradation. This is corroborated by our finding that increased cathepsin expression is accompanied with a decrease in collagen deposits in fibrotic lungs of bleomycin-challenged mice and supported by several other groups which reported a decrease in protein type I collagen and hydroxyproline contents in curcumin treated fibrotic lungs of rats [1-3].

Second, increased cathepsin expression may directly control TGF-β1 concentration in tissues by proteolysis. We have recently demonstrated that CatK deficiency in lung is associated with increased expression levels of TGF-β1 and that CatK is a potent TGF-β1 degrading protease [9]. Here we show that a cathepsin inhibitor increased TGF-β1 concentrations in fibroblasts by 100% suggesting a direct regulation of TGF-β1 concentration by cathepsin activities which will consequently affect proliferation and migration rates of lung cells. It has been demonstrated that abnormal repair and deregulated wound healing partly result from abnormal fibroblast proliferation and migration [11,36] and is stimulated by tissue factors such as collagen [37], PGDF [38], and TGF-β1 [39,40]. The degradation of collagen and TGF-β1 by CatK may thus provide stimulation signals to slow down proliferation, migration, and wound healing. Consequently, the administration of the cysteine protease inhibitor, LHVS, significantly increased the wound closure rate in a fibroblast scratch assay (Figure 4C) which was inhibited by the curcumin-mediated overexpression of cathepsins.

Interestingly, the increase in cathepsin expression by curcumin is greater under the condition of adding a fixed amount of exogenous TGF-β1 to the culture media (Figure 5D) than in the presence of bleomycin (Figure 2A and 2B) which has a TGF stimulatory effect. TGF-β1 itself has a suppressing effect on cathepsin expression (Figure 5D). This might be explained by the curcumin-mediated expression of a potent TGF-β1 degrading cysteine protease such as CatK [9] which is likely to deplete the amount of exogenously added TGF-β1. In the case of bleomycin stimulation, TGF-β1 will be continuously produced by the HFL-1 cells and thus its anti-cathepsin expression effect remains stronger.

The third effect of increased cathepsin expression is likely related to the apoptosis rates in fibrotic lungs. Wound healing and balancing the deposition of extracellular matrix largely depend on decreasing the resistance of fibroblasts to apoptosis [41]. Inadequate fibroblast and myofibroblast apoptosis may lead to the formation of fibrotic lesions [42]. Here, we demonstrated that curcumin increases apoptosis rates in fibrotic lung tissues and HFL-1 cells. This was evidenced by the dose-dependent increase of apoptotic markers such as of caspase-3 and the Bax/Bcl-2 ratio (Figure 6) and is in line with the finding that curcumin downregulates Bcl-2 in non-small cell lung cancer cells [43]. There are several potential pathways which may be responsible for curcumin induced fibroblast apoptosis. One pathway might be again related to TGF-β1. High levels of TGF-β1 have been shown to promote fibroblast resistance to apoptosis [44] whereas low levels of TGF-β1 may sensitize fibroblast to apoptosis. As discussed above the upregulation of cathepsin expression by curcumin leads to an increased degradation of TGF-β1 and thus would increase apoptosis rates. This is corroborated by the finding that curcumin increased the expression of caspase-3 and Bax/Bcl-2 in our experiments. Furthermore, when LHVS was employed as an antagonist to cysteine cathepsins activities in curcumin treated and bleomycin stimulated HFL-1 cells, the expression of proapoptotic markers was reduced. The cleavage of the Bcl-2 family member Bid by cathepsins has been proposed as a possible mechanism of cathepsin-mediated apoptosis [45].

The *in vivo *efficacy results were obtained after oral application of curcumin (200 mg/kg/d). Similar outcomes in terms of collagen deposition and/or TGF expression were observed by other authors using oral applications of curcumin in rats [2,3,46], whereas a recent study in mice reported efficacy only after *i.p*. administration of curcumin [1]. We looked into changes of ECM deposition based on Masson trichrome staining and immunohistochemical analysis of cathepsin and TGF-β1 expression whereas Smith and coworker [1] studied the inflammatory cell content, airspace, and hydroxyproline content. The hydroxyproline content as a marker of collagen accumulation revealed a trend of decrease by approximately 10% though not significant after 21 days of treatment in the Smith study [1]. Our data reflect a reduction in total collagen content by 18% after 14d and 32% after 28d. It is likely that an *i.p*. administration of curcumin would have resulted to more dramatic differences in our experiment as well.

In summary, our data provide evidence that cathepsin expression is increased in response to curcumin treatment in bleomycin challenged mice and HFL-1 cells. The elevated levels of cathepsins may directly promote ECM degradation, apoptosis and a decrease in TGF-β1 expression which indirectly affects fibroblast proliferation and thus ECM production, cell migration and apoptosis. This study adds to the increasing evidence that curcumin may represent a potentially effective drug for the treatment of human lung fibrosis.

## Competing interests

The authors declare that they have no competing interests.

## Authors' contributions

DWZ designed and carried out experiments, analyzed data and wrote the paper, CH and CY carried out all mouse experiments and analyzed the lung tissue samples, RJL performed some of the wound healing experiments, JW and JN conceived the mouse experiments and analyzed data, DB conceived experiments, analyzed data and wrote the paper. All authors read and approved the final manuscript.
